# Development of Quorum-Based Anti-Virulence Therapeutics Targeting Gram-Negative Bacterial Pathogens

**DOI:** 10.3390/ijms140816570

**Published:** 2013-08-09

**Authors:** Song Buck Tay, Wen Shan Yew

**Affiliations:** Department of Biochemistry, Yong Loo Lin School of Medicine, National University of Singapore, 14 Medical Drive, Singapore 117599, Singapore; E-Mail: bchtsbt@nus.edu.sg

**Keywords:** quorum sensing, anti-virulence, Gram-negative, quorum quenching, bacterial communication, *N*-acylhomoserine lactone, bacterial pathogens, disruption, inhibition

## Abstract

Quorum sensing is a cell density-dependent signaling phenomenon used by bacteria for coordination of population-wide phenotypes, such as expression of virulence genes, antibiotic resistance and biofilm formation. Lately, disruption of bacterial communication has emerged as an anti-virulence strategy with enormous therapeutic potential given the increasing incidences of drug resistance in pathogenic bacteria. The quorum quenching therapeutic approach promises a lower risk of resistance development, since interference with virulence generally does not affect the growth and fitness of the bacteria and, hence, does not exert an associated selection pressure for drug-resistant strains. With better understanding of bacterial communication networks and mechanisms, many quorum quenching methods have been developed against various clinically significant bacterial pathogens. In particular, Gram-negative bacteria are an important group of pathogens, because, collectively, they are responsible for the majority of hospital-acquired infections. Here, we discuss the current understanding of existing quorum sensing mechanisms and present important inhibitory strategies that have been developed against this group of pathogenic bacteria.

## 1. Introduction

Bacterial quorum sensing has attracted significant research interest since its initial discovery in the marine bacterium, *Vibrio fischeri*, almost four decades ago [1]. Recent progress includes the identification of new quorum sensing systems and a broader understanding of how bacteria organize collective action. One of the main reasons behind this gathered interest is the importance of quorum sensing in bacterial pathogenesis [2]. Quorum sensing regulates the expression of virulence genes through intercellular communication; this is mediated by quorum molecules and receptors in a density-dependent manner [3]. The apparent advantage of such interactions is the ability to coordinate population-wide phenotypes for effective host colonization and disease progression. Naturally, significant efforts have been focused on quorum sensing disruption, due to its role as a “master switch” in virulence control. Blocking communication can potentially prevent bacterial pathogenesis [4]. In terms of recent advancement, quorum sensing disruption is one of the emerging anti-virulence strategies against bacterial infections [5].

An anti-virulence approach is relevant in the current context, as antibiotics are losing their efficacy, and many bacteria are becoming multi-drug resistant (MDR) [6]. Although fundamentally effective against bacterial infections, antibiotics exert considerable selection pressure, as they target growth processes, such as nucleic acid replication and cell wall biosynthesis [7]. The eventual growth arrest and cell death can be followed by rapid expansion of resistant subpopulations, making subsequent treatment difficult or impossible [8]. However, it is the rate at which bacteria gain resistance that is alarming; these therapeutic agents were only in clinical use for just over half a century, and incidences of resistance have rapidly surfaced [9]. Over-prescription of antibiotics is the primary cause, as clinicians underestimated the long-term effects of these drugs. Faced with the risk of losing this treatment method, antibiotic prescription became better regulated and usage was often reserved for severe conditions [10]. Consequently, there are now limited treatment options for many infections, as bacterial evolution has outpaced drug development [11]. New anti-bacterial strategies are therefore required, and methods that aim to disable pathogenesis can prevent further development of drug resistance.

**Figure 1:**
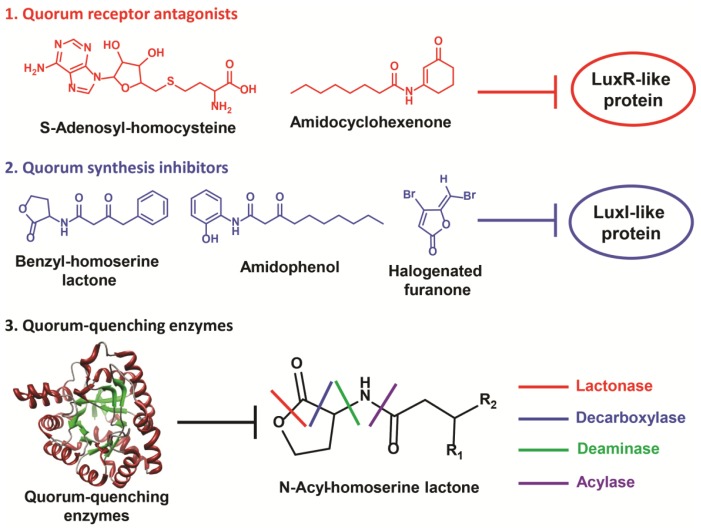
Anti-virulence strategies to disrupt bacterial quorum circuits.

A quorum-based method can provide several approaches for virulence disruption (Figure 1). In general, this is achieved via impeding the interaction between a quorum molecule and its cognate receptor. More importantly, quorum sensing is not essential for growth; hence, its disruption is unlikely to affect primary metabolic pathways necessary for survival [4]. Since fitness is not compromised, there is no associated selection pressure for the bacteria to evolve and acquire resistance against such treatments [12]. This is an advantage over traditional antibiotic methods, as bacterial pathogenesis can be mitigated without running the risk of immunity development; in addition, targeting bacterial quorum sensing also results in the attenuation of bacterial biofilm formation and the associated resistance to antibiotics [13,14]. In such cases, disruption of this signaling event can interfere with inherent bacterial protective mechanisms. With the recent understanding that quorum sensing is present in most bacteria species, this method presents great promise in the fight against bacterial pathogens.

Gram-negative bacterial pathogens account for the majority of hospital-acquired infections, resulting in extensive mortality and burden on global healthcare systems [15]. Reports have indicated that hospital-acquired infections complicate treatments and lead to an increasing risk of death. In a study conducted on intensive care unit (ICU) patients, a higher risk of infection was associated with longer ICU stays, and the mortality of infected patients doubled as compared to uninfected patients [16]. *Pseudomonas aeruginosa* and *Klebsiella pneumoniae* are known to be highly problematic in the ICU milieu, due to their affiliation with a multitude of nosocomial infections. More recently, some *Enterobacter* species, *Escherichia coli* and *Acinetobacter baumannii*, were added to this group as associated complications in bloodstream, surgical site and urinary tract infections continue to rise [17]. The severity of hospital-acquired infections is contributed by pathogens developing resistance against a wide variety of drugs. Certain strains of *P. aeruginosa*, *K. pneumoniae* and *A. baumannii* that are resistant to all antibiotics, also known as pan-drug resistant (PDR), have already been identified and are associated with considerable mortality [18]. Extended-spectrum β-lactamase (ESBL)-producing *Enterobacter* and *E. coli* are also proving very hard to treat, due to acquired resistance against broad spectrum drugs, such as cephalosporins, carbapenems and even non-β-lactams, such as fluoroquinolones [19,20]. Collectively, this group of Gram-negative bacterial pathogens account for the majority of nosocomial life-threatening infections; the challenge is on the research community to find new and more effective agents or methods to mediate the biomedical situation.

Given the current issue of antibiotic resistance and optimism in anti-virulence strategies, considerable work has been done to explore quorum sensing disruption in clinically important pathogens. This review focuses on Gram-negative bacterial pathogens and aims to provide a detailed account of quorum sensing regulation and strategies for the future advancement of anti-virulence therapeutic development.

## 2. Quorum Sensing in Gram-Negative Bacteria

### 2.1. Classical AHL- and AI-2-Mediated Quorum Sensing

**Figure 2:**
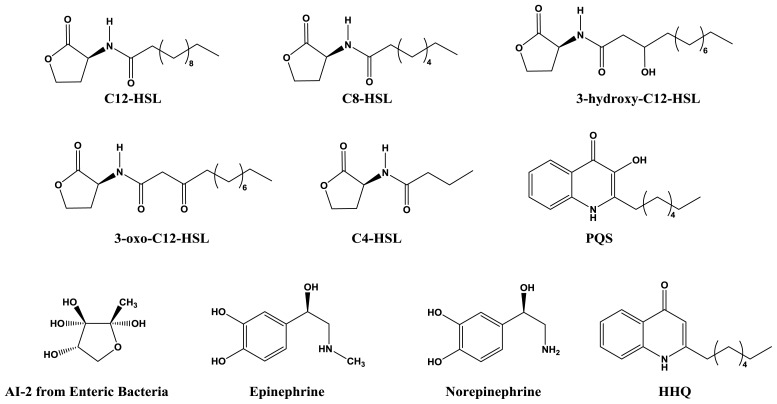
Chemical structures of quorum molecules.

The canonical quorum sensing system in bacteria consists of two main components: the quorum molecule and the receptor. *N*-Acyl homoserine lactones (AHLs) are the most common quorum molecules (Figure 2) used by Gram-negative bacteria, typically produced by synthases homologous to LuxI from *V. fischeri*. Chemical derivatives of AHLs vary in terms of acyl chain length and saturation state, as well as oxidation status at the third carbon position. A variety of acyl chains, ranging from four to 18 carbons in length, is linked to an invariant homoserine lactone ring via an amide bond. These quorum molecules, after synthesis, are mostly membrane permeable and are free to accumulate in both the intracellular and extracellular environment [21]. Since AHL concentrations are determined by diffusion, a high population cell density is required for sufficient intracellular accumulation [22]. Above a critical threshold concentration, AHLs binds to cognate receptors to form activated complexes.

Homology is also observed in AHL receptors that belong to the LuxR family of transcriptional regulators. Briefly, AHL binding relieves an inhibitory effect on the DNA binding motif, and the receptor complex is mobilized to drive target gene expression [23]. In certain bacteria, such as *Vibrio harveyi*, AHLs can also mediate transcription through a signaling cascade. An accumulation of AHLs produced by the LuxM synthase is detected by the corresponding LuxN receptor located on the cell membrane; receptor-ligand interaction results in a change in the phosphorylation state of the receptor, leading to the expression of downstream transcriptional activators [24,25]. Meanwhile, bacteria can have more than one AHL synthase/receptor pair. In species with multiple quorum networks, each set may be associated with different virulence phenotypes, though there is a certain amount of redundancy [26]. In any case, AHLs are used only by Gram-negative bacteria for quorum signaling and could be important for intraspecies communication.

Autoinducer-2 (AI-2) is another class of quorum molecules utilized by Gram-negative bacteria for cell-to-cell communication. However, AI-2 is not unique to Gram-negative bacteria, and it is believed to be involved in interspecies communication [27]. Bacteria with the AI-2 quorum sensing pathways can respond to both native and exogenous quorum molecules, even those produced by Gram-positive bacteria. This is particularly evident in *V. harveyi*, where two sensor strains (coupled to the expression of bioluminescence) are commonly used to detect AI-2 from other species [28]. 5′-Methylthioadenosine/S-adenosylhomocysteine nucleosidase (MTAN or Pfs) and LuxS are two essential enzymes required for the sequential synthesis of the AI-2 precursor, 4,5-dihydroxy-2,3-pentanedione (DPD) [29]. Upon formation, DPD is energetically unstable and undergoes spontaneous chemical rearrangement to generate a variety of DPD derivatives. Subsequent cyclization reactions would give rise to an active pool of AI-2 compounds that can exist in equilibrium [30,31]. The AI-2 pathway exists in many bacteria; to date, LuxS homologues have been identified in 537 of the 1,402 bacterial genomes that have been sequenced [32].

In contrast, the identification and categorization of AI-2 receptors is less extensive and is limited to a handful of bacterial species. Currently, the two most well-characterized AI-2 receptors are LuxP from *V. harveyi* and LsrB from *Salmonella typhimurium*; the crystallographic structures of these receptors have been determined [33]. Using the AI-2 system in *V. harveyi* as an example, LuxP receptors are typically periplasmic binding proteins with an affinity for the membrane-associated sensor kinase LuxQ. The activated LuxPQ induces a transition from kinase to phosphatase activity and production of transcriptional activators is upregulated via a series of signal transduction events [34,35]. In addition, LsrB-type receptors have also been identified in members of the Enterobacteriaceae, Rhizobiaceae and Bacillaceae families, due to their AI-2 binding abilities [36]. Since non-LuxP and LsrB producing bacteria also exhibit responses towards AI-2, there must be alternative receptors present in these bacteria as yet to be discovered. From understanding of the current literature, AI-2 is implicated in a wide range of bacterial phenotypes, namely biofilm formation, cell motility, conjugation and virulence factor production [26,37]. However, its contribution may not be limited to pathogenesis, as AI-2 quorum sensing is also known to influence metabolism and bacterial fitness to various extents [38].

### 2.2. Alternative Quorum Sensing Pathways in Gram-Negative Bacteria

Other pathways (Table 1) that regulate quorum sensing in Gram-negative bacteria include the 2-heptyl-3-hydroxy-4(1H)-quinolone (*Pseudomonas* quinolone signal [PQS]), 3-hydroxytridecan-4-one (cholera autoinducer-1 [CAI-1]), *cis*-11-methyl-2-dodecenoic acid (diffusible signal factor [DSF]) and autoinducer-3 (AI-3). The PQS pathway exists in *P. aeruginosa* and the operon, *pqsABCDE*, controls the initial production of the PQS precursor, 2-heptyl-4(1H)-hydroxyquinoline (HHQ), followed by a subsequent oxidative hydroxylation reaction by PqsH at the 3′ carbon position to convert HHQ to PQS [39,40]. Though PQS is used only by *P. aeruginosa* for quorum signaling, HHQ is used by other species in the *Pseudomonas* family and some species of *Burkholderia* for communication [41]. Both quorum molecules interact with the transcriptional regulator, PqsR, to mediate gene expression.

In the CAI-1 pathway, CqsA in *V. harveyi* and *Vibrio cholerae* are responsible for quorum molecule biosynthesis, and the downstream effect is a consequence of interaction with the sensor kinase, CqsS [26,42]. Signals are passed through a relay cascade involving components of the previously described AI-2 system in *Vibrio* species. Interestingly, in *V. cholerae*, CAI-1 favors the production of virulence factors when present at low concentrations; at higher CAI-1 concentrations, the expression of virulence factors is repressed [56].

The DSF quorum sensing pathway was first described in the plant pathogen, *Xanthomonas campestris* pathovar *campestris*; quorum molecule production is dependent on RpfF, an enoyl-CoA hydratase-related protein, and RpfB, a long-chain fatty acyl CoA ligase [57]. In a typical two component signaling model, RpfC functions as the receptor and an activated regulator component, RpfG, is known to degrade the second messenger molecule cyclic di-GMP to low levels intracellularly; this results in the synthesis of virulence factors and motility [58]. Recent studies have identified *rpf* gene cluster homologs in others species of *Xanthomonas*, *Xylella fastidiosa* and the human pathogen, *Stenotrophomonas maltophilia* [59,60]. Variable forms of DSF were also detected in *Burkholderia* species, and *B. cepacia* utilizes more than one type of DSF [61]. Although the relevance of having multiple DSF in one organism is still undefined, it is believed to be involved in intraspecies or interspecies communication [62]. This was demonstrated in *P. aeruginosa*, whereby these non-DSF producing bacteria were able to sense DSF signals to modulate their own behavior [63].

The AI-3 quorum sensing pathway proposed by Sperandio *et al.* is probably the least characterized communication network in Gram-negative bacteria. From enteric microbial flora studies, the QseC/B two component system is recognized as the sensor for AI-3, though the structure and gene responsible for AI-3 production remain unknown [64,65]. Epinephrine and norepinephrine have been found to activate the QseC/B system; hence, it is likely that AI-3 could resemble these hormones. Currently, AI-3 signaling has been described in *E. coli* and *S. typhimurium*, while subsequent studies on gastrointestinal bacteria could reveal further details about this pathway [49].

### 2.3. Quorum Sensing Mechanisms Used by Clinically Relevant Gram-Negative Pathogens

#### 2.3.1. Pseudomonas aeruginosa

*P. aeruginosa* is one of the most common human pathogen, and it is associated with a wide range of hospital-acquired infections, particularly with cystic fibrosis and burn patients. Due to the enormous clinical relevance of *P. aeruginosa*, its quorum sensing network is very well characterized and it is often used as a model organism for studies in the field [66]. Quorum sensing in *P. aeruginosa* generally exists as a complex hierarchical organization, where significant cross-talk between multiple pathways leads to a coordination of a significant number of genes [67]. To date, four quorum sensing systems have been described in the bacterium.

The *las* and *rhl* systems, identified much earlier, share similarities with the *lux* system from *V. fischeri*. AHL synthesis in both systems is mediated by LuxI-type synthases, LasI and RhlI, for production of *N*-(3-oxododecanoyl)-l-homoserine lactone (3-oxo-C12-HSL) and *N*-butanoyl-l-homoserine lactone (C4-HSL), respectively. These quorum molecules are recognized by their corresponding receptors, LasR and RhlR, when threshold quorum concentration is attained. In combination, the *las* and *rhl* systems regulate up to 353 genes or six percent of the *P. aeruginosa* genome; hence, a majority of *P. aeruginosa* quorum sensing research has focused on these two systems [43,68]. PQS is the third quorum molecule used by *P. aeruginosa*, and genomics studies have shown that 141 genes can be differentially controlled by the *pqs* system, suggesting a role in global regulation [69]. Production of PQS can be upregulated by the *las* system, although the *rhl* system mediates a negative effect [70]. In addition, the *pqs* system also auto-regulates its own production and promotes RhlIR expression [71,72]. Consequently, this results in a negative feedback loop that limits the synthesis of PQS. Likewise, both *rhl* and *las* systems mediate their own production, while the latter is also known to positively enhance the *rhl* system [73,74]. Despite its role as a positive regulator of adjacent pathways, the *las* system is not known to receive any feedback from both *rhl* and *pqs* systems.

In addition to the three quorum sensing systems, *P. aeruginosa* contains another LuxR-type receptor, QscR, for transcription modulation. The apparent distinction of QscR from the other two LuxR-type receptors is the lack of an associated synthase gene in the genome [44]. Without a cognate ligand pair, QscR is known to utilize 3-oxo-C12-HSL produced by LasI for signal activation [45]. The receptor also has an affinity for longer chain AHLs, and its promiscuity towards a variety of ligands indicates possible conduct in interspecies communication [75]. Based on earlier studies, QscR has a repressive effect on other quorum sensing systems, while the converse impact remains relatively uncharacterized [76]. From a transcription profiling study, QscR regulates overlapping genes with LasR and RhlR, though the “orphan” receptor also separately controls a distinct subset of genes [77].

#### 2.3.2. *Acinetobacter baumannii* and *Klebsiella pneumoniae*

Along with *P. aeruginosa*, *A. baumannii* and *K. pneumoniae* are categorized as the “ESKAPE” bugs, a group of problematic nosocomial pathogens that have evolved to escape the lethal actions of antibiotics [17]. As the threat of these emerging pathogens escalates, new drugs are constantly being tested for suitable anti-bacterial properties. However, quorum sensing perspectives in these two pathogens are still rather limiting. In *A. baumannii*, *N*-(3-hydroxydodecanoyl)-l-homoserine lactone (3-hydroxy-C12-HSL) is the only quorum molecule identified, though mass spectrometry quantitation suggested that other AHL molecules are also present at significantly lower amounts [78]. AbaI is the LuxI-type synthase responsible for 3-hydroxy-C12-HSL production, and signal detection is by its cognate AbaR receptor [51]. In addition, the *aba* system influences biofilm formation in the bacterium, thereby reducing susceptibility to antibiotics and enhancing species survival [79]. More recently, interspecies interaction between *A. baumannii* and *P. aeruginosa* was investigated to determine the effects of co-location in hospital settings: biofilm formation, bacterial growth and virulence of *A. baumannii* were not affected by metabolites produced by *P. aeruginosa*. Instead, biofilm formation was augmented when a heterologous pool of AHLs from both species was used, suggesting that complementary quorum interactions were responsible for symbiotic co-existence [80].

In *K. pneumoniae*, two quorum sensing systems have been described in the literature. The AI-2 quorum molecule was, for many years, the only recognized regulator of cell-to-cell communication in the bacterium. Synthesis of AI-2 and maturation of biofilm, through upregulation of lipopolysaccharide production, were essentially *luxS*-dependent [55,81]. Likewise, *luxS* transcription is sensitive to pH, glucose and boracic acid concentrations in the environment [82]. As for the subsequent AHL-mediated mechanism, it was discovered more recently from a dorsal human tongue profiling study. High resolution mass spectrometry analyses revealed that *N*-octanoylhomoserine lactone (C8-HSL) and *N*-3-dodecanoyl-l-homoserine lactone (C12-HSL) are the AHL molecules produced by *K. pneumoniae* [54]. Unfortunately, the specific receptor component was not determined in both mechanisms. In addition, hypervirulent strains of *K. pneumoniae* can enhance inherent virulence characteristics by releasing a siderophore-related quorum molecule to fully exploit iron deposits in the surrounding environment [83].

#### 2.3.3. *Enterobacter* spp. and *Escherichia coli*

*Enterobacter* spp. exists in the microbiota of soil, water, food and gastrointestinal tracts of animals and humans. Certain strains have gained resistance against antimicrobial agents and are recognized as increasingly important human pathogens in recent years. *E. aerogenes* and *E. cloacae* are among the two most prominent pathogens in the genus, with serious implications in respiratory and urinary tract infections [84]. Despite growing concerns with nosocomial and community risks, little is known about quorum control and pathogenesis in this group of bacteria. Outcomes of quorum sensing in *Enterobacte*r spp. were mainly revealed through food-associated studies. Earlier reports have shown that *Enterobacter* spp. isolated from chilled salmon and raw milk can produce a detectable amount of AHLs [85,86]. The quorum molecule was confirmed to be C12-HSL by high resolution mass spectrometry in a subsequent dorsal human tongue study [52]. In species that do not produce AHLs, such as *E. cloacae* GS1, a LuxR homolog, SdiA, has been detected. Similar to the QscR receptor from *P. aeruginosa*, SdiA is an “orphan” receptor that functions as a transcriptional regulator. The SdiA receptor in *E. cloacae* GS1 is sensitive to exogenous AHLs, and it has been found to negatively regulate bacterial adhesion and biofilm formation [53]. This result was corroborated by another study using a different strain of *E. cloacae* [87]. Furthermore, AI-2-mediated quorum sensing has also been suggested to play a role in intercellular communication within *Enterobacter* spp. Although the AI-2 quorum molecules have yet to be identified, Lsr-type receptors have been found in strains of *E. cancerogenus*, *E. cloacae* and *E. mori* [47].

Three quorum sensing circuits have been described in *E. coli* strains, namely SdiA, AI-2 and AI-3-mediated systems. With the exception of the AI-3 system, the other two circuits have been relatively well characterized. As mentioned, SdiA is an “orphan” LuxR homologue without a corresponding LuxI synthase [46]. Nevertheless, the *E. coli* SdiA receptor binds AHLs produced by other bacterial species, and the activated complex is believed to coordinate cell division, virulence and antibiotic resistance [46,88–90]. Similar to *Enterobacter* spp., SdiA also downregulates biofilm formation in *E. coli* and could serve to antagonize the effects of other quorum regulators [91]. In the parallel AI-2 system, AI-2 synthesis is controlled by the *luxS* gene, while signal binding, transport and modulation are encoded by the *lsr* operon. Briefly, AI-2 is delivered into the cell via the Lsr transporter, followed by LsrK-mediated phosphorylation [48]. LsrR is a repressor of the *lsr* operon and itself, however, phosphor-AI-2 can bind specifically to LsrR as an antagonist [92]. Subsequently, phosphor-AI-2 can be modified by LsrF and LsrG to effect further downstream regulation [93]. Overall, the *lsr* operon can be stimulated by the cyclic AMP (cAMP)-cAMP receptor complex [94]. The third AI-3 system, the QseC/B system, is mainly described in enterohemorrhagic *E. coli* (EHEC), and structural analysis of AI-3 has suggested that it is an aromatic compound [49]. In the presence of AI-3 or its analogous activators, the QseC/B system is mobilized to promote flagella formation and motility in the bacterium [50].

With multiple quorum sensing systems in *E. coli*, it is unlikely that each exist independently without any interaction. Recently, an EAL domain protein was found to facilitate interaction between the SdiA and AI-2 systems through cAMP regulation. A knockdown of *sdiA* and *ydiV* (the gene encoding for the EAL domain protein) led to a decrease in intracellular cAMP levels and subsequent inhibition of the AI-2 system [95]. Although the nature of the specific interplay between the EAL domain protein and cAMP is still unknown, the possibility to effect indirect regulation of a parallel quorum sensing system using downstream response elements was clearly demonstrated. Besides, the AI-2 system can also influence cellular motility under the control of the AI-3 system. This cross-system effect was found to be mediated by the quorum sensing regulator, MqsR, which interacts with the adjacent QseC/B system [96].

## 3. Quorum Sensing Disruption Strategies in Gram-Negative Bacteria

There are three essential processes for bacterial quorum sensing: quorum molecule synthesis, extracellular accumulation of quorum molecules and ligand/receptor-mediated activation of downstream processes. Therefore, quorum disruption strategies generally adopt three common approaches: inhibition of quorum molecule synthesis, inhibition of quorum molecule/receptor interaction and degradation of quorum molecules (Figure 1). Though many quorum inhibitors were developed for broad spectrum effects, it is important to note that quorum sensing is largely unique to each bacterial species (Table 2). In some cases, there is more than one quorum sensing system present, and a combination of inhibition strategies may be necessary to prevent pathogenesis. In the next section, we will discuss quorum sensing disruption methods used in some clinically significant Gram-negative pathogens. The review is not meant to be exhaustive; instead, it is written to highlight the current important inhibitory developments against these pathogenic bacteria. *P. aeruginosa* will be used as an example for elaboration of inhibition methods, since such studies on the pathogen have been comprehensive. In pathogenic bacteria where quorum inhibition efforts are still lacking, possible approaches will be discussed.

### 3.1. Quorum Sensing Disruption in *Pseudomonas aeruginosa*

#### 3.1.1. Inhibition of AHL Detection

As a model organism for quorum sensing research, *P. aeruginosa* has been used extensively for various inhibition studies with modest success. Among the methods explored, interference with signal detection has been one of the most commonly reported strategies. Inhibitors and analogues of native quorum molecules can recognize and bind to receptor elements yet prevent subsequent signaling through the formation of an inactive ligand-receptor complex. For the AHL group of quorum molecules, the length and saturation state of the acyl tail can be critical for binding affinity and activity [105]. AHL molecules with a conserved lactone ring, but modified acyl tail, have been shown to exhibit preferential binding and antagonism with LasR receptor, and the presence of a 3-oxo moiety was noticed to favor LasR interaction [105]. In addition, other AHL molecules with variation at the lactone ring have also been synthesized and found to be inhibitory towards LasR and RhlR receptors [106]. Collectively, antagonists of LuxR-type receptors have included both natural and synthetic analogues of native quorum molecules. Screening of natural compounds is likely to be more unbiased, and selected antagonists may display greater structural diversity. Recently, Yang *et al*. identified a structural analogue of *N*-decanoyl-l-homoserine lactone (C10-HSL) with inhibitory effects against *P. aeruginosa* [97]. The compound, *N*-decanoyl-l-homoserine benzyl ester (C2), repressed transcription of *las* and *rhl* genes, while concomitantly downregulating production of rhamnolipid and swarming motility in the *P. aeruginosa* PAO1 strain. On the other hand, synthetic derivatives of quorum molecules are usually designed to be specific and retain considerable properties to the corresponding template compounds. Blackwell *et al*. have synthesized many non-native AHL analogues and systematically evaluated their inhibitory efficacies in several Gram-negative pathogenic bacteria over the years. Among a selection of potential antagonists, the compound, D15, with an aromatic substitution on the acyl chain, exhibited potent quorum sensing inhibition against *P. aeruginosa* [98]. The LasR receptor was the target in the study, and the addition of antagonists generally negated elastase B production. In a subsequent study by the same group, new antagonists that target the QscR receptor of *P. aeruginosa* have also been discovered [99]. The most potent antagonists were identified from compounds containing an *N*-benzoyl substitution on the acyl chain; however, the associated phenotypic response of the antagonism has yet to be elucidated. More recently, Morkunas *et al*. explored the strategy of using synthetic AHL mimics with significant feedback, suggesting that antagonists of *P. aeruginosa* LuxR-type receptors can originate from a diverse structural background [107].

Crystal structures of receptor bound to its quorum molecule can provide useful information on key interactions between the two components. In the case of *P. aeruginosa*, with the availability of 3D structural data, computational docking was used to screen for inhibitors against its LuxR-type receptors. Through this method, several compounds antagonistic to LasR and RhlR were successfully identified and their activity validated *in vitro* [108]. In addition, the crystal structure of LasR has facilitated the development of electrophilic probes that can target the ligand binding pocket of the receptor [100]. The probes bind covalently to the Cys79 residue within the pocket, specifically inhibiting the LasR receptor. The ability of an inhibitor to specifically target a pathogen is crucial in realizing its full therapeutic potential. Consequently, agonists or antagonists that are specific for certain receptors become therapeutic leads. Ganguly *et al*. exploited the specificity of C4-HSL analogues and showed that these compounds can be used to deliver covalently bound antibiotics to disrupt biofilm formation in *P. aeruginosa* [101]. Incidentally, C4-HSL is not exclusive to *P. aeruginosa*, and the antibiotic conjugate could be non-specific in mixed culture conditions. Similarly, LuxR-type receptors share significant homology; therefore, antagonists of the LasR, RhlR and QscR receptors can potentially have inhibitory effects on analogous receptors in other bacterial pathogens.

#### 3.1.2. Inhibition of AHL Accumulation

Since quorum sensing is a density-dependent mechanism, prevention of signal accumulation is one of the most intuitive and viable approaches for disruption. Due to their catalytic prowess, enzymes have been postulated to be effective agents that degrade or inactivate quorum molecules (of Gram-negative bacteria). There are three groups of enzymes known to act on AHLs, namely acylases, lactonases and oxidoreductases. Acylases hydrolyze the amide bond in AHLs, releasing the homoserine lactone and fatty acyl chain, while lactonases target the hydrolysis of the ester bond in the lactone ring. Oxidoreductases, however, do not hydrolyze the quorum molecule, but rather modify it to an inactive form [109–111]. Each of these classes of enzyme has been shown to target AHLs produced by *P. aeruginosa*.

The AiiD protein isolated from *Ralstonia* strain is one of the first acylase used against AHLs; this enzyme was able to completely degrade 3-oxo-C8-HSL, 3-oxo-C10-HSL and 3-oxo-C12-HSL, respectively, within three hours of incubation [109]. When AiiD protein was expressed in *P. aeruginosa* PAO1, there was a corresponding decrease in elastase and pyocyanin production, as well as a reduction in swarming and nematode paralyzing ability. In a separate study, an AiiA homolog expressed in the same *P. aeruginosa* strain was able to prevent the accumulation of both AHLs secreted by the bacteria, thereby leading to a decrease in associated virulence factor production [112]. The AiiA protein, belonging to the metallo-β-lactamase superfamily, is the first lactonase found to degrade AHLs, although later studies have identified other members with similar activity. Likewise, the effect of using lactonases in combination with antibiotics was studied by means of biofilm microplate assays. Interestingly, the addition of lactonases increased antibiotic susceptibility: less gentamicin and ciprofloxacin were required for eradication of biofilms formed by MDR *P. aeruginosa* as compared to lactonase-free treatment [13]. In contrast, oxidoreductase activity against *P. aeruginosa* AHLs was characterized more recently. The protein bpiB09 was identified through metagenomic analysis as an NADP-dependent reductase, suggesting that AHLs are not the native substrates of the enzyme [113]. Nevertheless, bpiB09 was shown to act on 3-oxo-C12-HSL, and the ensuing quorum inhibition led to the reduction of pyocyanin and biofilm formation. In spite of *P. aeruginosa*’s susceptibility to AHL quenching enzymes, the bacterium is also capable of producing its own enzymatic quenchers. The *P. aeruginosa* associated PA0305 protein was identified to be an acylase with activity against long chain AHLs, including 3-oxo-C12-HSL, the cognate quorum sensing molecule in the bacteria [114]. In particular, PA0305 probably controls quorum molecule levels in the local environment. In addition, bacteria can be thought to produce quorum quenching enzymes to inactivate non-native signal molecules (from competing bacteria) for niche colonization [115].

Besides enzymes, antibody-mediated quorum inactivation has also been demonstrated. Using a monoclonal antibody XYD-11G2 fused to a hapten containing a squaric monoester monoamide motif, Marin *et al.* showed that the antibody was able to “neutralize” 3-oxo-C12-HSL and disrupt pyocyanin production in *P. aeruginosa* [116]. Furthermore, the hapten linker functions as mimic of the AHL acyl chain and can thus be developed for greater specificity. Antibodies can also sequester AHLs, rendering them inactive. Kaufmann *et al.* showed that (a stoichiometric amount of) monoclonal antibody RS2-1G9 can readily sequester 3-oxo-C12-HSL and protect macrophages from *P. aeruginosa* [117]. Overall, the immunopharmacotherapeutic approach is a promising strategy for the design and development of novel quorum inhibitors/quenchers to combat bacterial virulence.

#### 3.1.3. Inhibition of the *pqs* System

As compared to the well-studied *las* and *rhl* systems, there are noticeably less reports on *pqs* inhibition studies. Nonetheless, inhibition of the *pqs* system is important for attenuation of quorum sensing and virulence in associated bacterial species. All three quorum inhibition approaches mentioned prior (inhibition of quorum molecule synthesis, inhibition of quorum molecule accumulation and inhibition of ligand/receptor interaction) have been explored for this system. Inhibition of quorum detection is probably the most direct approach, and Lu *et al.* were the first to discover antagonists against the receptor, PqsR, using a ligand-based drug design method. Antagonists 18 and 19 were particularly effective, as pyocyanin levels can be diminished with low micromolar concentrations of the compounds [102]. With the characterization of the PqsR X-ray crystallographic structure, crucial ligand-receptor interactions can be determined, paving the way for the future design of novel PqsR antagonists [118]. The structural similarity between PQS and a natural substrate, 3-hydroxy-2-methyl-4(1H)-quinolone, has prompted the use of a relevant dioxygenase enzyme (Hod) for quorum inhibition. As expected, the enzyme Hod was able to convert PQS to *N*-octanoylanthranilic acid and carbon monoxide, preventing the accumulation of PQS in *P. aeruginosa* cultures. Consequently, there was a decrease in PQS-regulated virulence determinants, lectin A, pyocyanin and rhamnolipids [119]. In addition to the first two approaches, inhibition of PQS synthesis can be achieved by using anthranilate analogues (incidentally, these analogues are precursors of PQS). Calfee *et al.* first reported inhibition of PQS synthesis using methyl anthranilate, with the resulting decrease in downstream elastase production in *P. aeruginosa* [120]. Subsequently, Lesic *et al*. also found that halogenated anthranilate had similar inhibitory effects, including a disruption of MvfR-dependent gene expression [121]. The MvfR pathway apparently plays an essential role in quorum sensing, as *P. aeruginosa* dissemination in the research model was significantly reduced. More importantly, these compounds restricted quorum sensing without affecting bacterial growth, hence decreasing the risk of resistance development.

#### 3.1.4. Inhibition by Natural Compounds

Besides the prior-mentioned candidates, natural compound libraries serve as another source of quorum inhibitors/quenchers. Plants, in particular, are known to produce highly complex molecules with a variety of medicinal or therapeutic properties. Many of these molecules, however, may be very difficult to synthesize chemically, due to their complicated chemical scaffolds; hence, direct extraction would be the method-of-choice to obtain these products. A significant number of plant-extracted compounds have already been discovered with bacterial quorum quenching activity. Most recently, Plyuta *et al*. have found phenolic plant extracts that are able to inhibit biofilm formation in the *P. aeruginosa* PAO1 strain when applied at high concentrations [122]. However, at sub-inhibitory concentrations, these compounds have an opposite effect and supported biofilm growth instead. Some of these compounds included vanillin, 4-hydroxybenzoic and gallic acids, which do not resemble the prototypical structure of AHLs. In addition, ellagic acid derivatives from *Terminalia chebula* and aqueous extracts from *Conocarpus erectus*, *Callistemon viminalis* and *Bucida buceras* also had reported inhibitory effects on quorum sensing of *P. aeruginosa* [123,124]. Ellagic acid derivatives disrupted expression of LasR and RhlR, while concomitantly decreasing virulence factor production and enhancing the sensitivity of *P. aeruginosa* PAO1 biofilm towards tobramycin treatment. The later study was conducted in a *Caenorhabditis elegans* model, and the aqueous extracts prevented gut infection in 60% of nematodes and reduced mortality by 50%. Importantly, these extracts were effective without causing host toxicity and could be useful for further development as therapeutics. In addition to plant-derived compounds, natural compounds derived from insects and marine life have also been found, though these examples will not be elaborated in this review [125,126]. In view of the extensive ecological diversity in the natural world, many useful compounds have yet to be discovered, and studies into novel and uncharacterized natural extracts would undoubtedly expand the lexicon of effective compounds beyond the current known libraries.

### 3.2. Quorum Sensing Disruption in *Escherichia coli*

*E. coli* is a significant nosocomial pathogen, and as a model organism for life science research, its quorum sensing network is well described. Sufficient understanding of the quorum sensing architecture may be correlated to greater inhibition efforts, as this was evident when compared against species that are less studied. In *E. coli*, representative work has been done on the existing quorum sensing pathways. This part of the review will highlight some of the quorum sensing disruption efforts in each of the pathways described in this bacterium.

As mentioned earlier, MTAN is involved in the synthesis of the AI-2 precursor DPD by converting *S*-adenosylhomocysteine (SAH) to *S*-ribosylhomocysteine (SRH) in the SRH biosynthetic pathway [9]. Typically, MTAN inhibitors can disrupt SRH biosynthesis and, thus, prevent AI-2 formation. Gutierrez *et al.* have shown that transition analogues of MTAN (5′-Methylthio-DADMe-Immucillin-A, 5′-ethylthio-DADMe-Immucillin-A and 5′-butylthio-DADMe-Immucillin-A, respectively) can function as inhibitors of AI-2 synthesis, and their corresponding picomolar range dissociation constants indicated high binding affinity to the associated enzymes [127]. The study also highlighted the feasibility of blocking quorum molecule production and, at the same time, emphasized the importance of MTAN in AI-2 synthesis. On the other hand, analogues of DPD can also be used for quorum sensing inhibition in the bacterium. *Guo et al.* demonstrated that ester derivatives of DPD analogues introduced into *E. coli* can be hydrolyzed subsequently inside the cells to reveal a biologically active diol functional group for quorum sensing disruption [128]. Interestingly, when identical DPD analogues were delivered into *S. typhimurium*, a similar disruption effect was not observed, suggesting that selective quorum sensing modulation could possibly be achieved between closely related species using this type of ester-linked compound. The reason for such selectivity remains unclear; however, the authors suggested that it could be due to differential analogue permeation and esterase sensitivity. In addition to MTAN and DPD analogues, enzymatic quenching of the AI-2 signal is another method that can be used to disrupt this pathway. In the following enzyme-mediated example, the native bacterial phosphorylation machinery, LsrK, was retooled to inactivate its own AI-2 molecule [129]. Under native conditions, LsrK is the kinase responsible for phospho-AI-2 generation; phosphor-AI-2 then activates a series of *lsr*-controlled genes. When Roy *et al.* used LsrK to phosphorylate AI-2 molecules *in vitro* and added phospho-AI-2 exogenously to *E. coli* cultures, quorum sensing was significantly inhibited. This observation suggested an alternative function for LsrK: it could downregulate *lsr*-controlled genes when present in an extracellular context; this effect can also be attributed to the impermeability of membranes to phosphor-AI-2 molecules.

AI-3 related quorum sensing inhibition in *E. coli* has also been reported in the literature. Rasko *et al.* showed that a small organic molecule, *N*-phenyl-4-[(phenylamino)thioxomethyl]amino-benzenesulfonamide, termed LED209, was able to attenuate expression of QseC-associated genes to reduce virulence in EHEC strains [103]. More recently, Vikram *et al.* also described the use of isolimonic acid and ichangin as potent inhibitors of Type III secretion systems (TTSS) and biofilm formation in a QseC- and QseA-dependent manner [130]. In addition, there were further accounts of natural compounds that interfered with *E. coli* quorum sensing. Using *Melia dubia* seed extracts, Ravichandiran *et al.* found that the crude content was able to reduce biofilm and swarming motility in uropathogenic *E. coli* (UPEC) by targeting the SdiA-mediated quorum sensing pathway [131]. Notably, honey when delivered at low concentrations can also inhibit biofilm formation in EHEC. Sugar moieties in honey, such as glucose and fructose, were likely to be responsible for the observed inhibitions [132]. Biofilm reduction was attributed to the repression of AI-2 quorum sensing and virulence gene expression [133]. In other studies, halogenated furanones have been recognized as inhibitors of quorum sensing, due to their structural similarity to bacterial AHLs. Givskov *et al.* were one of the first groups to report the inhibitory effects of brominated furanones on bacterial species [134]. Later, the effects of brominated furanones were also shown in *E. coli* by means of a comparative gene expression study. The objective of investigation, however, focused on the AI-2-mediated pathway, and it was suggested that furanones can also alter AI-2 signaling post-transcriptionally to downregulate biofilm formation and motility phenotypes [135].

### 3.3. Quorum Sensing Disruption in *Klebsiella pneumoniae, Enterobacter* spp. and *Acinetobacter baumannii*

According to a report by the Centers for Disease Control and Prevention, *K. pneumoniae*, *A. baumannii and Enterobacter* spp. collectively accounted for more than 13 percent of hospital-acquired infections in the two-year period of 2009 to 2010 [136]. Moreover, highly resistant strains of these bacteria can kill up to 40% of infected patients, prompting the healthcare industry to revisit abandoned drugs for “last resort” treatments. Colistin, for example, is a polymyxin antibiotic useful against most Gram-negative bacilli; however, due to the associated risk of neurotoxicity and nephrotoxicity, the drug became obsolete 20 years ago. Yet, in the last decade, colistin prescription became more common against MDR pathogens, although some instances of resistance have already been reported [137,138]. Given the clinical importance, it is surprising that not much work has been done to address the virulence of these pathogens (*vis-à-vis* quorum inhibitions). Studies related to *K. pneumoniae*, *A. baumannii and Enterobacter* spp. have been limited, and the methods developed are unlikely to be useful for complete attenuation of quorum sensing in these bacteria. For example, Derakhshan *et al.* were the only group to show that essential oil from cumin seeds reduced biofilm formation in *K. pneumoniae*. From scanning electron microscopy images, it was evident that the capsular layer of *K. pneumoniae* was affected, possibly accounting for the observed two-fold decrease in biofilm formation [139]. However, there was no direct evidence to show that this reduction was mediated via quorum sensing pathways (although it is known that quorum sensing regulates the biofilm formation phenotype in the bacteria).

In the case of *Enterobacter* spp., Reis Ponce *et al.* have found that AiiA lactonase lowered cell numbers during early biofilm formation by *Enterobacter cloacae* [87]. The effect of quorum quenching enzymes was later corroborated by Kim *et al.* in an anti-biofouling study, which showed that *Enterobacter* microbial populations were reduced by half in treated membrane bioreactors (MBRs) [140]. Proteomic analysis further confirmed that flagellin and outer membrane protein expressions were downregulated, possibly resulting in a decrease in MBR biofilm mass. For *A. baumannii*, disruption of quorum sensing has been limited to the use of non-native AHLs and antagonists containing aromatic acyl groups [104]. Interestingly, non-native d-AHLs were also strong antagonists of the AbaR receptor, with increased potency against L-AHLs analogues, although d-AHLs are predicted to be less effective against LuxR-type receptors. In any case, the antagonists identified were successful in inhibiting AHL-mediated quorum sensing and its subsequent surface motility and biofilm formation. Most recently, Saroj *et al*. also found that streptomycin at sub-inhibitory concentrations is able to inhibit quorum sensing in *A. baumannii* [141]. Streptomycin (and not other antibiotics, such as gentamicin and myomycin) downregulated transcription of two quorum sensing genes, *abaI* and A1S_0112, resulting in the corresponding decrease in 3-oxo-C12-HSL production. It was proposed that streptomycin could act as an antagonist of AbaR and prevent positive feedback activation of *abaI* by functional 3-oxo-C12-HSL-AbaR complexes. In addition to this study, several others have also showed that sub-inhibitory concentrations of antibiotics can positively or negatively modulate quorum sensing, in contrast to bactericidal effects observed at high concentrations: tobramycin was found to induce biofilm formation in *E. coli* and *P. aeruginosa* when applied at concentrations three-fold below the minimal inhibitory concentration (MIC) [142]. It has been suggested that antibiotics could act as intermicrobial signaling agents, with roles in maintaining homeostasis in bacterial biofilm formation, motility and TTSS, instead of their widely regarded function as biological adversaries [143]. Such a hypothesis provides a refreshing evolutionary explanation for the observed relationship between antibiotics and quorum sensing in these bacteria.

In spite of the above-mentioned examples, studies related to quorum inhibition of *K. pneumoniae*, *A. baumannii and Enterobacter* spp. are still rather limited. The current lack of quorum inhibitors for clinically significant Gram-negative pathogens is a mounting concern, especially with the continued evolution of resistance mechanisms and the prevalence of MDR strains. When “last resort” drugs, such as colistin and tigecycline lose their efficacies, there will be even less treatment options available against MDR species. Fortunately, at least for the moment, we may not have to look beyond existing strategies, as many Gram-negative pathogens share similar quorum sensing mechanisms. Generic pathways and methods can be exploited and used against affiliated species. As mentioned, MTAN inhibitors were able to disrupt DPD formation and prevent AI-2 synthesis in *E. coli* strains. Since the AI-2 biosynthetic pathway is conserved in many bacterial species, inhibition of AI-2 production can target bacteria with the existing quorum sensing system [32]. In addition, MTAN is also involved in the synthesis of AHLs through the conversion of 5′-methylthioadenosine (MTA) to 5-methylthioribose (MTR). MTA is a by-product of polyamine synthesis from S-adenosylmethionine (SAM), and MTAN is important for the salvage of methionine for SAM regeneration [144]. Currently, potent MTAN inhibitors with picomolar range efficacies have been determined, and there is potential that MTAN inhibitors could be important anti-bacterial agents for suppression of AHL synthesis by disrupting SAM supply [145]. Alternatively, the enoyl-acyl carrier protein reductase (FabI), which is important for AHL acyl chain formation, can be a target of inhibition. The role of FabI in AHL synthesis was demonstrated by Hoang *et al.* using FabI mutants, and the group have further shown that triclosan at low concentrations successfully inhibited FabI by almost 50 percent [146]. It is palpable that such approaches can be applicable to pathogens that use LuxI-type of AHL synthases to potentially disrupt quorum sensing.

**Figure 3:**
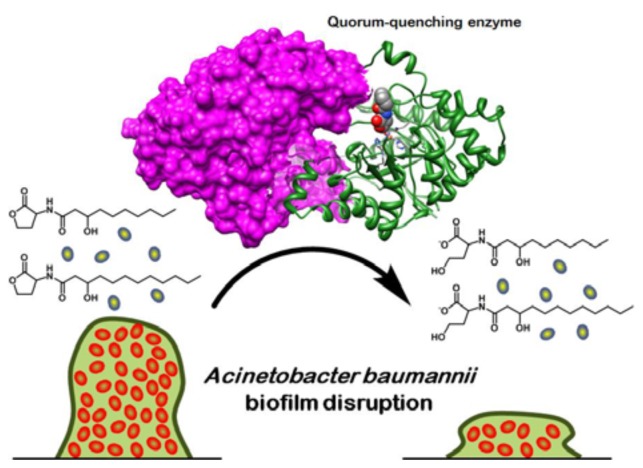
Disruption of biofilm formation in *Acinetobacter baumannii* using engineered quorum-quenching lactonases.

Quorum-quenching enzymes are another noteworthy group of quorum inhibitors, as many enzymes have already been characterized, and there is substantial literature coverage in terms of hydrolyzable AHLs. Furthermore, bacteria share many common AHLs; hence, enzymatic degradation of these signaling molecules might be useful in broad-spectrum control of virulence in various pathogenic bacteria [147]. Enzymes usually exhibit substrate preference, as seen in the case of AiiD acylase, where catalytic specificity is towards longer chain AHLs [109]. However, exceptions do exist among AHL acylases: the AiiC acylase from *Anabaena* sp. is promiscuous and is able to degrade AHLs ranging from four to 14 carbons in acyl chain length. In addition, the enzyme does not discriminate against any particular substitution group on the acyl chain, suggesting a probable involvement in broad range signal interference within a complex microbial community [148]. Likewise, there are also broad-spectrum quorum-quenching enzymes from the lactonase group. The QsdA lactonase from *Rhodococcus erythropolis* is a distinct example, where AHLs with acyl chain lengths ranging from six to 14 carbons can be inactivated [149]; similar to the reactivity of AiiC, the oxidation state at position three of the acyl chain is not discriminated against by the QsdA enzyme. In a more recent study, Chow *et al.* described an engineered GKL lactonase from *Geobacillus kaustophilus* with enhanced catalytic activity against AHLs ranging from six to 12 carbons in acyl chain length [150]. Notably, the engineered GKL has improved hydrolytic activity of up to 72-fold (catalytic efficiency, *k_cat_/K_M_*) against longer chained AHLs and a newly acquired C4-HSL activity (the wild-type enzyme had no detectable lactonase activity against C4-HSL). The improved lactonase was identified through directed evolution methods, illustrating the utility of this approach for engineering novel substrate specificities into existing quorum quenching enzyme scaffolds. The authors also demonstrated the translational potential of the study by using the engineered quorum-quenching lactonase to disrupt biofilm formation in *A. baumannii* (Figure 3). From crystal violet assays and confocal imaging results, the biomass of *A baumannii*-associated biofilms was significantly reduced; this work represents the first successful account of biofilm disruption using recombinant quorum-quenching enzymes [151]. The study also highlighted the utility of using enzymes with broad AHL specificity to treat infectious diseases associated with a variety of Gram-negative bacteria (including bacterial pathogens with multiple AHL quorum systems).

## 4. Quorum-Quenching Considerations and Implications

Interference with bacterial virulence, via quorum-quenching, promises to be “tolerance-free”, as the approach is not bactericidal in nature [12]. Indeed, bacteria viability is usually not affected when quorum sensing is blocked, and there has been considerable success since interest gathered in the field almost a decade ago. Even though quorum sensing inhibitors have been shown to be effective in preventing virulence factor expression, reducing biofilm formation and altering membrane permeability, bacteria do persist after treatment. Therefore, to achieve their therapeutic potential, quorum sensing inhibitors may have to be used concurrently with antibiotics for both anti-virulence and anti-bacterial effects. Certain inhibitors are known to increase susceptibility to antibiotics, although the combined treatment was rarely tested [13]. In addition, due to the complexity of bacterial quorum circuits (some bacteria have more than one quorum sensing system) the disruption of one quorum system often does not effectively prevent the expression of virulence factors. There are four distinct quorum regulatory circuits in *P. aeruginosa* that are interconnected by regulator-mediated feedback mechanisms. Such redundancy allows for compensatory responses by other systems, and shutting down the entire complex network poses a great challenge: in the absence of a *las* system in *P. aeruginosa*, RhlR was still able to control LasR-specific functions, providing a testament to the difficulty in addressing quorum-mediated pathogenesis by the bacteria [152]. In addition, the production of 3-oxo-C12-HSL and PQS were not affected, indicating a complicated hierarchy in quorum circuitry in the human pathogen. On a different note, quorum sensing can negatively regulate virulence phenotypes, as evidenced by the SdiA-mediated system in *E. cloacae* GS1 [53]. Hence, the disruption of quorum sensing in such exceptional systems would instead increase bacterial virulence.

An anti-virulence approach represents an anticipated solution to PDR bacterial infections, yet such optimism has to be periodically reviewed and tempered with caution. Recently, Defoirdt *et al*. challenged the assumption that quorum sensing disruption is resistance-free and suggested that fitness of bacteria can be affected through variability in quorum sensing core genes [153]. In retrospect, support for this resistance-free concept was gathered through a credible research effort conducted in laboratory-based conditions. However, conditions could differ significantly *in vivo*, and it was highlighted by Martinez *et al*. that evaluation of fitness should be made in models that better reflect actual infection and treatment settings [154]. Certainly, bacteria within a host environment will be subjected to a very different set of selective pressures (such as those imposed by the host immune system). As mentioned earlier, bacteria can compensate for partial quorum sensing inhibition by using alternative or redundant circuits for virulence [152]. Similarly, quorum sensing-deficient mutants can also exploit signals and metabolites produced by quorum sensing-proficient variants for virulence, since quorum sensing is fundamentally a cooperative behavior that is beneficial to the community [155]. The emergence of social “cheaters” has already been reported by Sandoz *et al*. in their study where *P. aeruginosa lasR* mutants ceased production of quorum regulated factors and take advantage of those produced by their proficient neighbors [156]. Taking an alternative perspective, quorum sensing-insensitive mutants capable of producing quorum regulated factors can likewise interfere with quorum inhibition efforts. A proof-of-concept study was carried out by Mellbye *et al.* illustrating that inhibition-resistant subtypes (“cooperators”) were able to profiteer from inhibition-sensitive subtypes when the quorum regulated product was secreted into the extracellular milieu (“public goods”). More importantly, mutual sharing in this case does not necessarily lead to a selective advantage for “cooperators”, and the enrichment of resistant subtypes can be mitigated. Nevertheless, quorum-quenching efficiency will be compromised when resistant subtypes are present in the treatment population. On the other hand, if the quorum regulated product is intracellular (“private goods”), inhibition of quorum sensing could favorably select for resistant subtypes, as the “products” generally cannot be shared [157]. Essentially, this study highlighted an often neglected consideration in quorum inhibition research, in that the discrimination of “public” or “private” goods is crucial in preventing the emergence of bacterial resistance to quorum inhibitors.

## 5. Conclusions

Research efforts in the past few decades have contributed significantly to the understanding of the mechanisms of bacterial communication. Due to the rapid occurrence of antibiotic resistance, recent focus was placed on inhibitory studies to address the therapeutic potential for the healthcare industry. Disruption of quorum sensing is widely regarded as an attractive anti-virulence approach, as this strategy is non-bactericidal and, therefore, would not generate the unwanted selective pressure for resistance. A variety of quorum disruption strategies (with varied success) against pathogenic bacteria have been described, including the inhibition of quorum molecule synthesis, accumulation and detection. Gram-negative bacteria represent a significant group of human pathogens, as they are responsible for a majority of hospital-acquired infections. Despite successful efforts against some of the more common pathogens, there remain a number of less-studied (and understood) bacterial pathogens waiting for effective therapeutic intervention: there have been limited reports on quorum sensing disruption in pathogens, such as *A. baumannii*, *K. pneumoniae* and the *Enterobacter* species. Along with the studies on quorum inhibition, there is also concern about bacterial resistance against anti-virulence methods. In fact, there is increasing evidence of quorum quenching treatments leading to a rise in resistant bacterial phenotypes. Further studies are necessary to determine the molecular mechanisms of mutations that confer the resistant bacterial phenotypes and if the selection of such mutations is by chance and would confer advantages in fitness. Nevertheless, future studies in the therapeutic development of anti-virulence strategies should proceed with care and caution to avoid the undesired fate currently associated with antibiotic development.

## Figures and Tables

**Table 1:** Quorum systems of selected Gram-negative bacteria.

Bacteria	Receptor(s)	Synthase(s)	Quorum molecule(s)	References
*V. harveyi*	LuxN	LuxM	3-hydroxy-C4-HSL	[24]
	LuxP	LuxS	AI-2	[33]
	CqsS	CqsA	CAI-1	[42]
*P. aeruginosa*	RhlR	RhlI	C4-HSL	[43]
	LasR	LasI	3-oxo-C12-HSL	[43]
	QscR	NA	3-oxo-C12-HSL	[44,45]
	PqsR	PqsABCD, PqsH	PQS, HHQ	[40,41]
*E. coli*	SdiA	N.A.	3-oxo-C8-HSL	[4,46]
	LsrB	LuxS	AI-2	[47,48]
	QseC	- -	AI-3/ Epinephrine/Norepinephrine	[49,50]
*A. baumannii*	AbaR	AbaI	3-hydroxy-C12-HSL	[51]
*Enterobacter* spp.	- -	- -	C12-HSL	[52]
	SdiA	NA	- -	[53]
	LsrB	LuxS	AI-2	[47]
*K. pneumonia*	- -	- -	C8-HSL	[54]
	- -	- -	C12-HSL	[54]
	LsrB	LuxS	AI-2	[47,55]

N.A.: Not applicable; - -: Not yet characterized.

**Table 2:** Quorum sensing inhibitors.

Inhibitor	Structure	Target bacteria	Target	Effect/value	References
*N*-Decanoyl-L-homoserine benzylester (C2)	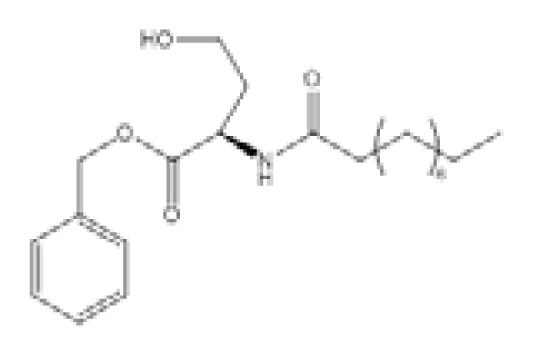	*P. aeruginosa*	LasR, RhlR expression	LasR - at 100 μM C2: 29.67% reduction RhlR - at 100 μM C2: 28.20% reduction	[97]
Compound D15	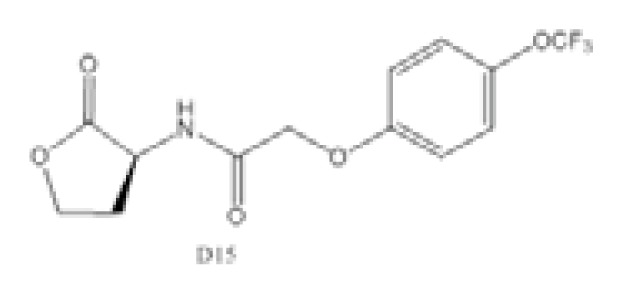	*P. aeruginosa*	IC_50_	4.67 μM	[98]
Compound Q9	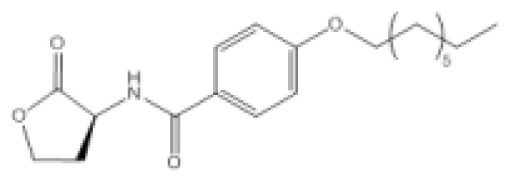	*P. aeruginosa*	IC_50_	11 nM	[99]
Isothiocyanate-13 (itc-13)	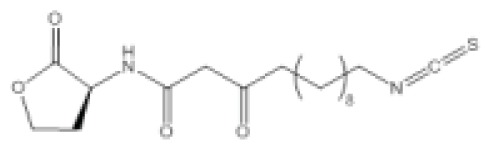	*P. aeruginosa*	IC_50_	45.2 μM	[100]
QS0108-ciprofloxacin conjugate	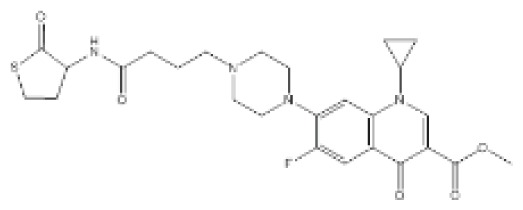	*P. aeruginosa*	MIC	50 μM	[101]
Compound 18	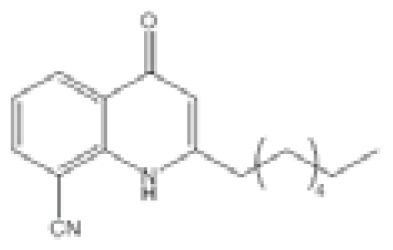	*P. aeruginosa*	IC_50_	259 nM	[102]
Compound 19	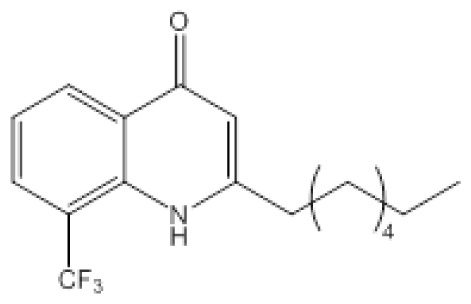	*P. aeruginosa*	IC_50_	54 nM	[102]
*N*-Phenyl-4-{[(phenylamino)thioxomethyl]amino}-benzenesulfonamide (LED209)	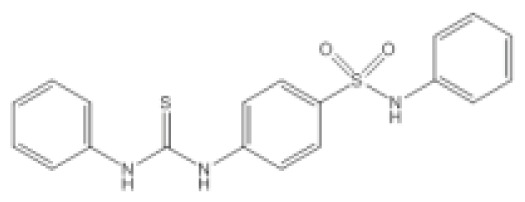	*E. coli*	IC_50_	<10 μM	[103]
Compound C8	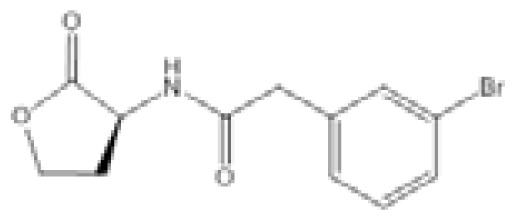	*A. baumannii*	IC_50_	5.06 μM	[104]
Compound C11	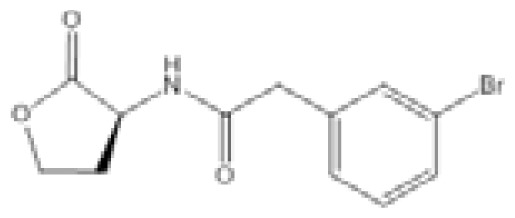	*A. baumannii*	IC_50_	2.32 μM	[104]
